# Fungal Pulmonary Coinfections in COVID-19: Microbiological Assessment, Inflammatory Profiles, and Clinical Outcomes

**DOI:** 10.3390/biomedicines13040864

**Published:** 2025-04-03

**Authors:** Petrinela Daliu, Iulia Bogdan, Ovidiu Rosca, Monica Licker, Livia Claudia Stanga, Elena Hogea, Delia Berceanu Vaduva, Delia Muntean

**Affiliations:** 1 Doctoral School, “Victor Babes” University of Medicine and Pharmacy Timisoara, Eftimie Murgu Square 2, 300041 Timisoara, Romania; petrinela.daliu@umft.ro; 2 Department of Infectious Disease, “Victor Babes” University of Medicine and Pharmacy Timisoara, Eftimie Murgu Square 2, 300041 Timisoara, Romania; iulia-georgiana.bogdan@umft.ro (I.B.); ovidiu.rosca@umft.ro (O.R.); 3 Methodological and Infectious Diseases Research Center, Department of Infectious Diseases, “Victor Babes” University of Medicine and Pharmacy Timisoara, Eftimie Murgu Square 2, 300041 Timisoara, Romania; 4 Discipline of Microbiology, Faculty of Medicine, “Victor Babes” University of Medicine and Pharmacy Timisoara, Eftimie Murgu Square 2, 300041 Timisoara, Romania; licker.monica@umft.ro (M.L.); berceanu.delia@umft.ro (D.B.V.); muntean.delia@umft.ro (D.M.); 5 Microbiology Laboratory, “Pius Brinzeu” County Clinical Emergency Hospital, 300723 Timisoara, Romania

**Keywords:** COVID-19, pulmonary fungal infections, antifungal resistance, disease severity, clinical outcomes

## Abstract

**Background and Objectives:** Secondary pulmonary fungal infections in coronavirus disease 2019 (COVID-19) remain underexplored despite emerging reports linking them to heightened morbidity. Comorbidities, steroid use, and prolonged hospital stays can predispose patients to opportunistic fungi. This study aimed to evaluate the impact of fungal coinfection on inflammatory markers, disease severity, antifungal resistance profiles, and outcomes in hospitalized COVID-19 patients. **Methods:** This retrospective observational study enrolled 280 adults (≥18 years) with real-time polymerase chain reaction (RT-PCR)-confirmed COVID-19 admitted to a tertiary care center (January 2023–December 2024). Patients were divided into a COVID-19-only group (*n* = 216) and a COVID–fungal group (*n* = 64) based on bronchoalveolar lavage, sputum, and/or blood culture positivity for fungal pathogens. Inflammatory markers (C-reactive protein (CRP), procalcitonin, the neutrophil-to-lymphocyte ratio, and the systemic immune inflammation index) and severity scores (Acute Physiology and Chronic Health Evaluation II, CURB-65 score, and the National Early Warning Score) were measured. We assessed antifungal susceptibilities and recorded ICU admissions, ventilation, hospital length of stay, and mortality. **Results:** *Aspergillus fumigatus* (31.3%), *Candida albicans* (28.1%), *Cryptococcus neoformans* (7.8%), *Pneumocystis jirovecii* (6.3%), and *Mucorales* (6.3%) dominated; *Candida glabrata*, *Candida tropicalis*, and mixed infections were also noted. Multidrug-resistant (MDR) isolates or resistance to triazoles occurred in 25.0% of cultures. The COVID-19–fungal group showed significantly higher CRP (85.7 vs. 71.6 mg/L, *p* < 0.001), procalcitonin (2.4 vs. 1.3 ng/mL, *p* < 0.001), and APACHE II scores (18.6 vs. 14.8, *p* < 0.001). intensive-care unit admissions (39.1% vs. 19.9%, *p* = 0.004) and mechanical ventilation (26.6% vs. 10.2%, *p* = 0.01) were more frequent with fungal coinfection. Mortality trended at a higher rate (15.6% vs. 7.4%, *p* = 0.06). **Conclusions:** Pulmonary fungal coinfections intensify the inflammatory milieu, elevate severity scores, and lead to more frequent ICU-level interventions in COVID-19 patients. Early identification, guided by culture-based and molecular diagnostics, alongside prompt antifungal therapy, could mitigate adverse outcomes. These findings underscore the critical need for proactive fungal surveillance and rigorous stewardship in managing severe COVID-19 pneumonia.

## 1. Introduction

Although much attention has been focused on the direct pathophysiological effects of severe acute respiratory syndrome coronavirus 2 (SARS-CoV-2), secondary infections—particularly those involving fungi—are increasingly recognized as key drivers of hospital complications [1,2,3]. Fungal pathogens can exploit the immunological dysregulation and tissue damage inflicted by severe viral pneumonia [4,5,6]. Such coinfections may go underdiagnosed if clinicians assume that clinical deterioration stems solely from COVID-19 progression [7].

Notable fungal pathogens in hospitalized populations include *Candida albicans*, *Candida glabrata*, *Aspergillus fumigatus*, *Cryptococcus neoformans*, *Pneumocystis jirovecii*, and *Mucorales* species (*Mucor*, *Rhizopus*) [8,9,10]. Pulmonary involvement can manifest with similar signs to bacterial pneumonia or acute respiratory distress syndrome, including a new or persistent fever and worsening hypoxia [11,12]. In immunocompromised states or in patients receiving high-dose corticosteroids for COVID-19, these fungi may proliferate rapidly, posing substantial diagnostic and therapeutic challenges [13,14].

Beyond the respiratory tract, fungi can seed in distant sites. *Candida* species, for instance, frequently cause candidemia with potential dissemination to the eyes (endophthalmitis), central nervous system, or other organs [15,16]. *Aspergillus* can invade pulmonary vasculature, leading to infarctions and hemoptysis [17]. *Mucorales* may destroy infected tissue at a fulminant pace, especially in diabetic patients, raising the specter of mucormycosis that can extend from the sinuses to orbital or cranial structures [18]. Awareness of these complications is paramount, given their dire prognostic implications.

Inflammatory markers (C-reactive protein (CRP) and procalcitonin) and more nuanced indicators like the neutrophil-to-lymphocyte ratio (NLR) and systemic immune inflammation index (SII) can offer a window into infection severity in COVID-19 patients [19,20]. However, distinguishing purely viral-driven changes from those secondary to fungal coinfections can be difficult without dedicated diagnostic sampling. Clinical scoring tools such as the Acute Physiology and Chronic Health Evaluation II (APACHE II), CURB-65 (confusion, urea, respiratory rate, blood pressure, age), and the National Early Warning Score (NEWS) may aid in risk stratification, yet they do not differentiate between infection etiologies [21,22,23]. Comprehensive workups that include microbiological and radiological testing have become essential in identifying the etiology behind clinical deterioration.

Meanwhile, the rise in antifungal resistance is a growing concern, as resistance to triazoles in *Aspergillus*, echinocandin resistance in *Candida*, and difficult-to-treat strains of *Candida auris* have been reported internationally [24,25,26]. Empirical antifungal use could further drive resistance, just as broad-spectrum antibiotics fueled the spread of multidrug-resistant bacteria. Hence, stewardship programs must weigh the clinical urgency of early antifungal initiation against the epidemiological threat of overuse.

In this study, we aimed to compare a cohort of patients hospitalized for COVID-19, some of whom developed pulmonary fungal coinfections, against those without mycotic involvement. We investigated differences in inflammatory markers, disease severity, antifungal susceptibility patterns, and clinical outcomes, including ICU admissions, mechanical ventilation needs, and mortality. By delineating these relationships, we aim to inform clinical decision-making with the purpose of choosing the right antifungal in a timely manner.

## 2. Materials and Methods

### 2.1. Study Design and Setting

This retrospective cohort study was conducted at a tertiary care academic hospital in Timisoara, Romania (Eastern Europe), at the Victor Babes Hospital for Infectious Disease and Pulmonology, affiliated with the Victor Babes University of Medicine and Pharmacy in Timisoara, Romania. The study period extended from January 2021 to December 2024, encompassing the era during which SARS-CoV-2 variants continued to circulate. Institutional review board approval was obtained prior to data collection. The study protocol was approved by the Institutional Review Board, and follows the Helsinki guidelines for human research. All adult inpatients (≥18 years) with real-time polymerase chain reaction (RT-PCR)-confirmed COVID-19 were screened for inclusion.

### 2.2. Patient Selection and Group Allocation

Inclusion criteria were (a) age ≥ 18 years, (b) confirmed COVID-19 infection via RT-PCR from nasopharyngeal swabs, and (c) hospitalization for COVID-19 management. Exclusion criteria comprised patients with incomplete records or those transferred from outside facilities without baseline diagnostic information. All eligible participants were stratified into two groups: (1) COVID-19-only group (*n* = 216): No evidence of fungal infection upon admission or during hospitalization. Fungal cultures and antigen tests, if obtained, were negative. And a COVID-19–fungal group (*n* = 64): Laboratory-confirmed fungal infection via sputum or bronchoalveolar lavage culture, blood culture (for candidemia or other fungemia), or rapid antigen/molecular tests (for *Cryptococcus* or *Pneumocystis*). Clinically, these patients had radiological or laboratory findings suggestive of fungal disease.

### 2.3. Data Collection

Demographic and clinical information included age, sex, body mass index (BMI), and major comorbidities (hypertension, diabetes, chronic lung disease, chronic kidney disease, malignancy). Duration of diabetes (in years) was recorded to verify that it did not differ significantly between groups, minimizing its confounding effect. All relevant data were abstracted from electronic health records by trained medical staff. Inflammatory markers (CRP and procalcitonin), complete blood count parameters (including absolute neutrophils, absolute lymphocytes, and platelets), and albumin levels were recorded within 48 h of admission and periodically thereafter. NLR and SII were computed. Three severity scores—APACHE II, CURB-65, and NEWS—were calculated upon admission or at the point of clinical deterioration for patients transferred from wards to the ICU.

Respiratory sampling included sputum when available and bronchoalveolar lavage for patients requiring ICU care or for those with a high clinical suspicion of fungal pneumonia. Blood cultures were obtained in all febrile patients or those showing hemodynamic instability.

Antifungal susceptibility testing was performed using standardized broth microdilution methods. Yeast isolates were assessed according to CLSI M27-A3 guidelines, while filamentous fungi, including *Aspergillus* spp. and Mucorales, were tested following CLSI M38-A2 protocols. Minimum inhibitory concentrations (MICs) for azoles, echinocandins, and amphotericin B were determined and interpreted based on established CLSI or EUCAST clinical breakpoints, with isolates exceeding these thresholds classified as resistant. Multi-drug resistance was defined as resistance to two or more antifungal classes. For organisms such as *Pneumocystis jirovecii*, for which susceptibility testing is not routinely performed, treatment decisions were based on standard therapeutic protocols (e.g., TMP-SMX).

True infections were differentiated from mere colonization according to two positive cultures (sputum or BAL) and corroborating clinical/radiological findings suggestive of active disease (e.g., new or persistent infiltrates or increasing inflammatory markers). For Aspergillus, we relied on microbiological assays (galactomannan antigen testing) alongside imaging and patient risk factors (use of corticosteroids or immunosuppressants) to classify cases as invasive rather than colonizing. While a subset of non-intubated patients displayed less severe clinical courses, we believe these diagnostic measures appropriately separated asymptomatic colonization from genuine infections.

Routine bacterial cultures and serological testing (including procalcitonin trend analyses) were conducted to screen for potential bacterial co-infections. Although PCT can be less discriminatory for fungal infections, some patients with fungal disease did show elevated PCT, which in several instances aligned with superimposed bacterial pneumonia (e.g., positive bacterial sputum cultures) or systemic inflammation due to COVID-19. Where laboratory and clinical evidence indicated bacterial co-infection, targeted antibiotic therapy was initiated promptly, and such cases were statistically controlled for in our final analyses.

### 2.4. Statistical Analysis

Data were compiled using SPSS (v. 28, IBM Corp., Armonk, NY, USA). Numerical variables were expressed as the mean ± standard deviation. Between-group comparisons utilized Student’s *t*-test for normally distributed variables; non-parametric tests (Mann–Whitney U) were employed where appropriate. Categorical data (ICU admissions, mortality) were compared using chi-square or Fisher’s exact test. A *p*-value < 0.05 denoted statistical significance. Subgroup analyses by ICU status and antifungal resistance patterns further delineated risk factors and outcomes.

## 3. Results

### Patient Demographics

Table 1 presents the demographic and clinical characteristics of patients with COVID-19 and fungal superinfection (COVID-19–fungal, *n* = 64) compared to those with COVID-19 alone (COVID-19-only, *n* = 216). There was no significant difference in patients’ age among the two study groups, as the COVID-19–fungal group had a mean age of 59.7 ± 9.8 years versus 58.5 ± 10.2 years in the COVID-19-only group (*p* = 0.395) and a similar proportion of males (57.8% vs. 58.8%, *p* = 0.869). Body mass indices (27.6 vs. 27.3 kg/m^2^, *p* = 0.650) and the prevalence of hypertension (56.3% vs. 56.0%, *p* = 0.974), diabetes mellitus (29.7% vs. 29.6%, *p* = 0.997), chronic kidney disease (15.6% vs. 13.4%, *p* = 0.657), COPD (14.1% vs. 12.0%, *p* = 0.667), malignancy (9.4% vs. 6.5%, *p* = 0.428), and smoking status (25.0% vs. 22.7%, *p* = 0.700) were comparable between the two groups. Additionally, the duration of diabetes was similar (8.2 vs. 8.0 years, *p* = 0.817). Notably, the time from symptom onset to hospital admission was longer for the COVID-19–fungal group (6.3 vs. 5.8 days, *p* = 0.059).

Table 2 details the distribution of fungal species and the occurrence of major complications among patients with COVID-19 and fungal superinfection (*n* = 64). The most prevalent fungal pathogen identified was *Aspergillus fumigatus*, accounting for 31.3% of cases, followed closely by *Candida albicans* at 28.1%. Other *Aspergillus* species and *Candida* species were present in 6.3% and 12.5% of patients, respectively. Additionally, *Cryptococcus neoformans*, *Pneumocystis jirovecii*, and *Mucorales* (including *Mucor* and *Rhizopus*) each accounted for 6.3% to 7.8% of infections, while mixed fungal species were rare, being observed in only 1.6% of cases. Regarding major fungal complications, *Aspergillus* invasive pulmonary infection was the most common, occurring in 12.5% of patients, followed by Candidemia (10.9%) and Cryptococcal pneumonia (7.8%). *Pneumocystis* pneumonia and Mucormycosis with tissue invasion were identified in 6.3% and 4.7% of patients, respectively, while *Candida* endophthalmitis was rare, being seen in 1.6% of cases.

Table 3 illustrates the antifungal susceptibility and resistance patterns among the fungal isolates identified in the study (*n* = 64). *Aspergillus fumigatus* was the most frequently isolated species (31.3%, *n* = 20) and exhibited a notable resistance to azoles, with 20% of isolates (4/20) showing resistance. Additionally, 10% of the *A. fumigatus* isolates were multi-drug resistant. Other *Aspergillus* species accounted for 6.3% (*n* = 4) of the isolates, with a higher azole resistance rate of 25% (1/4) and a multi-drug resistance rate also at 25%. *Candida albicans* constituted 28.1% (*n* = 18) of the isolates, demonstrating 11.1% resistance to azoles and 5.6% to echinocandins, while no resistance to amphotericin B was observed; one isolate (5.6%) was multi-drug resistant. In contrast, other *Candida* species made up 12.5% (*n* = 8) and showed no azole resistance, but 25% were resistant to echinocandins and 25% were multi-drug resistant. *Cryptococcus neoformans* (7.8%, *n* = 5) had a 20% resistance rate to azoles with no resistance to amphotericin B and no multi-drug resistance observed. Mucorales (6.3%, *n* = 4) typically exhibit resistance to azoles; among these, 25% were resistant to amphotericin B and 25% were multi-drug resistant. *Pneumocystis jirovecii* isolates (6.3%, *n* = 4) did not undergo standard antifungal susceptibility testing as trimethoprim–sulfamethoxazole (TMP-SMX) was utilized for treatment. Overall, the resistance rates were 14.3% for azoles, 9.5% for echinocandins, 1.6% for amphotericin B, and 9.5% for multi-drug resistance across all isolates.

Table 4 compares key inflammatory markers and select laboratory parameters between those with fungal coinfection and those without. Patients in the COVID-19–fungal cohort demonstrated consistently higher values for most inflammatory markers. The mean CRP in the COVID-19–fungal group was 85.7 mg/L, statistically higher than the 71.6 mg/L recorded in the COVID-19-only group (*p* < 0.001). Likewise, procalcitonin—often elevated in bacterial or more severe systemic infections—showed a nearly two-fold increase (2.4 vs. 1.3 ng/mL, *p* < 0.001). Elevated white blood cell counts (8.7 vs. 7.5 × 10^9^/L, *p* = 0.003) further point to a more intense immunologic activation. The neutrophil-to-lymphocyte ratio (6.4 vs. 4.9, *p* < 0.001) and systemic immune inflammation index (1185.3 vs. 968.7, *p* < 0.001) also markedly diverged, reinforcing that coinfected patients appear to have a more pronounced inflammatory response. Platelet levels trended at lower levels among the COVID-19–fungal group (221.3 vs. 236.6 × 10^9^/L, *p* = 0.08), although not significantly so. Albumin was reduced (31.5 vs. 34.0 g/L, *p* = 0.002).

**Figure 1:**
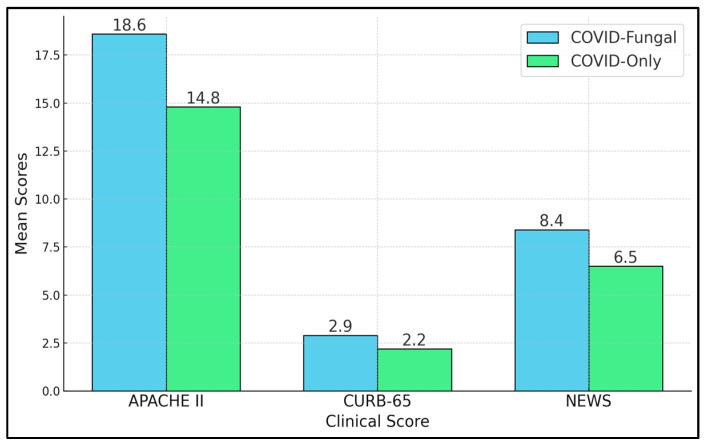
Clinical severity scores.

Table 5 and Figure 1 highlight three severity scores employed to estimate patients’ risk of deterioration. The COVID-19–fungal group exhibits substantially higher APACHE II scores (18.6 vs. 14.8, *p* < 0.001), indicating more severe systemic disease. The CURB-65 score, typically used for pneumonia prognostication, was also significantly greater in the COVID-19–fungal cohort (2.9 vs. 2.2, *p* = 0.001). CURB-65 factors include confusion, blood urea nitrogen, respiratory rate, blood pressure, and age; higher values correlate with an increased risk of inpatient mortality and a potential need for critical care. Meanwhile, NEWS provided a snapshot of acute deterioration risks based on vitals such as oxygen saturation, heart rate, and blood pressure. Here, again, coinfected patients showed more concerning levels (8.4 vs. 6.5, *p* < 0.001).

ICU admission rates were almost double in the COVID-19–fungal group (39.1% vs. 19.9%, *p* = 0.004), signifying that a substantial subset of those with pulmonary fungal involvement deteriorate and require advanced critical care interventions. Similarly, mechanical ventilation was needed in 26.6% of coinfected patients, more than twice the 10.2% incidence observed in the COVID-19-only group (*p* = 0.01). Although in-hospital mortality did not reach the conventional threshold for statistical significance (15.6% vs. 7.4%, *p* = 0.06), the near doubling of the death rate in the COVID-19–fungal group underscores the clinically relevant risk posed by fungal infections. Further, the mean hospital length of stay was prolonged by nearly three days (14.2 vs. 11.5, *p* = 0.003), as presented in Table 6.

**Figure 2:**
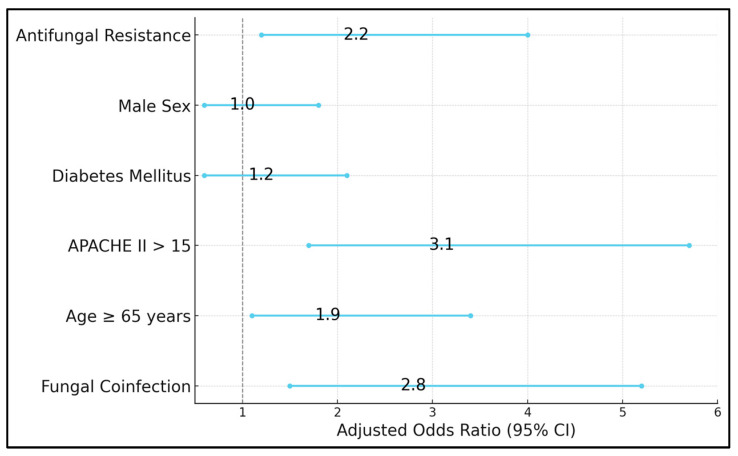
Risk factor assessment for ICU admission in COVID-19 patients.

Table 7 outlines a multivariable logistic regression model aiming to identify independent predictors for ICU admission among all 280 study participants. The presence of fungal coinfection emerges as a strong risk factor (adjusted OR 2.8, 95% CI 1.5–5.2, *p* = 0.002), indicating that, even when controlling for other variables like age and diabetes, patients with confirmed fungal involvement remain significantly more likely to require ICU-level care. Advanced age (≥65 years) also confers an elevated risk (adjusted OR 1.9, *p* = 0.02). APACHE II scores above 15 (OR 3.1, *p* < 0.001) retain their central significance, illustrating that the comprehensive physiological burden captured by this score correlates closely with ICU transfers. Interestingly, diabetes mellitus (OR 1.2, *p* = 0.53) does not emerge as a statistically significant predictor once fungal status and severity scores are included, suggesting that while diabetes may predispose patients to infection, it is the active presence of fungal disease and overall severity that predominantly drives ICU admissions. Of particular note, antifungal resistance (OR 2.2, *p* = 0.01) significantly influences ICU admission, reflecting how resistant infections complicate management and can precipitate clinical decline (Figure 2).

## 4. Discussion

The current study demonstrates that fungal coinfections in COVID-19 patients notably increase both the inflammatory burden and severity of illness. The elevated CRP, procalcitonin, NLR, and SII levels in the COVID-19–fungal group align with existing evidence that secondary fungal infections elicit a pronounced immune response [27,28]. Correspondingly, higher APACHE II, CURB-65, and NEWS scores underscore how coinfected patients experience more complex clinical courses [29,30]. Although limited in sample size, the near twofold increase in ICU admissions and mechanical ventilation among these patients signals a meaningful clinical impact that cannot be solely explained by viral pneumonia. Collectively, these findings corroborate earlier reports that opportunistic fungi—*Aspergillus*, *Candida*, *Cryptococcus*, *Pneumocystis*, and *Mucorales*—pose serious threats in vulnerable populations [31].

Our data highlight important aspects of the fungal epidemiology in COVID-19, *with Aspergillus fumigatus* and *Candida albicans* as the leading pathogens [32]. However, a noteworthy proportion of non-albicans *Candida* and *Cryptococcus* also emerged, in some cases demonstrating resistance to key antifungals. The detection of echinocandin-resistant *Candida glabrata* and amphotericin B–resistant *Mucorales* further complicates therapeutic decisions. In line with global observations, these resistant fungi can undermine empirical treatment strategies, making targeted therapy and antifungal stewardship critical [33]. The relatively high rate of candidemia, invasive aspergillosis, and even rarer entities like mucormycosis and cryptococcal pneumonia suggests that diagnostic protocols—such as bronchoalveolar lavage, fungal antigen testing, and blood cultures—should be implemented early in patients whose clinical course deviates from typical viral pneumonia [34].

This was previously reported in the study by Blaize et al. who assessed the occurrence of candidemia among severely ill COVID-19 patients admitted to five ICUs in France [35]. This retrospective analysis, conducted from March 2020 to January 2021, included 264 patients, predominantly male (70.5%) and immunocompetent (87.5%), with a significant portion (62.7%) receiving extracorporeal membrane oxygenation support. Despite extensive screening involving 4864 blood cultures and 975 beta-glucan tests, candidemia was identified in only 4.9% (13 patients) of the cohort, suggesting a relatively low risk of developing invasive candidiasis in this patient population. ICU mortality was not significantly impacted by the occurrence of candidemia, and there was a notable rate of unrelated positive beta-glucan tests (23.4%), indicating potential false positives and other mold infections.

Conversely, the study by Caciagli et al. [36] explored the association between COVID-19-associated invasive pulmonary aspergillosis (CAPA) and cytomegalovirus (CMV) reactivation in a cohort of 579 critically ill COVID-19 patients in Italy. Conducted prospectively from February 2020 to May 2022, the study identified CAPA in 16.6% of the patients, with a notable 41.7% of these patients experiencing CMV reactivation. Despite the high incidence of co-infection, there was no significant increase in 90-day mortality in patients with both CAPA and CMV reactivation compared to those with CAPA alone. This suggests a complex interplay between fungal and viral infections in critically ill COVID-19 patients that does not necessarily translate to higher mortality but does result in longer ICU stays and fewer ventilation-free days.

The absolute increase in fatality rates for coinfected patients warrants vigilance. Overlapping risk factors—age, baseline comorbidities, and immune dysfunction—likely compound the impact of SARS-CoV-2 infection. The logistic regression results pinpoint fungal coinfection and antifungal resistance as critical contributors to ICU admission, emphasizing how these infections aggravate the course of COVID-19 beyond well-known parameters like age or diabetic status [37,38]. Recognizing these multifactorial complexities, a thorough assessment of patients with persistent fevers, escalating inflammatory indicators, or non-resolving pneumonia should include fungal diagnostics. Earlier detection could enable prompt antifungal therapy, potentially curtailing the progression toward ICU care or mechanical ventilation.

Similar to earlier studies, in their study, Bhatt et al. [39] highlighted the exacerbated vulnerability of severe COVID-19 patients to opportunistic fungal infections like mucormycosis, driven by the significant reduction in T lymphocytes, CD4^+^ T, and CD8^+^ T cells observed in approximately 85% of these patients. The presence of lymphopenia emphasized this heightened risk, particularly for those on immunosuppressive treatments like corticosteroids and monoclonal antibodies, which although essential, can further compromise immune defenses. On a related note, the research conducted by Gold et al. [40] substantiated these observations by reporting an 8.5% annual increase in hospitalizations involving fungal infections during the COVID-19 pandemic in the United States. More alarmingly, their study found that COVID-19-associated fungal infections were linked with a dramatically higher in-hospital mortality rate of 48.5% compared to 12.3% in patients with non–COVID-19-associated fungal infections, underscoring the severe impact of fungal co-infections in COVID-19 patients.

In a similar manner, the meta-analysis by Liu et al. [41] established a strong association between mycotic infections and an increased risk of mortality in COVID-19 patients, identifying an odds ratio (OR) of 2.69 (95% CI: 2.22–3.26) for mortality and an OR of 2.28 (95% CI: 1.65–3.16) for the need for renal replacement therapy. These findings underscored the severe impact of fungal infections on the progression and outcomes of COVID-19, with particularly high odds ratios reported in Europe and Asia and *Candida* identified as the most perilous fungal strain. Similarly, the retrospective cohort analysis conducted by Zuniga-Moya et al. [42] focused on COVID-19-associated invasive fungal infections (CAIFIs) among intubated patients, revealing a 2.80% overall incidence of CAIFIs with *Aspergillus* and *Candida* as the predominant fungi. The study further quantified the impact on mortality, with hazard ratios (HRs) for CAIFIs caused by *Aspergillus* at 2.0 (95% CI: 1.8–2.2) and for Candida at 1.7 (95% CI: 1.5–1.9), indicating significant increases in all-cause mortality among these patients.

In a similar manner, the prospective study conducted by Negm et al. at Zagazig University Hospitals found a significant incidence of fungal coinfections in critically ill COVID-19 patients, with 32.8% diagnosed with such infections during their ICU stay [43]. *Candida* was the most prevalent fungus, affecting 24.1% of the cohort, followed by *Aspergillus* at 4.3%, and mucormycosis at 1.97%. Key risk factors associated with fungal coinfections included poor diabetic control and prolonged high-dose steroid use, with odds ratios indicating substantial increases in risk—14.1 (95% CI: 5.67–35.10) for steroid use and 14.57 (95% CI: 5.83–33.78) for multiple comorbidities. Similarly, the nationwide case–control study by Maeshima et al. [44], part of the J-RECOVER study group, highlighted the role of prolonged steroid therapy as a significant predictor of fungal infections in severe COVID-19 patients requiring mechanical ventilation. Their analysis identified a steady increase in the risk of fungal infections with each additional day of steroid use (OR per day increase: 1.01; 95% CI: 1.00–1.01). Both studies underscore the critical impact of immunosuppressive therapy and underlying comorbidities in the development of fungal infections among hospitalized COVID-19 patients, suggesting a need for careful management of such risk factors to mitigate associated complications.

Several limitations affect the generalizability and granularity of our results. First, this was a single-center study, capturing a local fungal epidemiology that may not reflect patterns in other regions or healthcare settings. Second, some fungi are notoriously difficult to culture; reliance on conventional culture methods may have led to underdiagnosis or delayed identification in cases where advanced diagnostics (e.g., next-generation sequencing) were unavailable. Third, while we adjusted for major confounders (age, comorbidities, disease severity), residual confounding by unmeasured variables cannot be ruled out—particularly regarding immunosuppressive medication regimens or the specific timing and choice of antifungal therapy. Fourth, the relatively modest sample size of the COVID-19–fungal group limited our ability to detect smaller differences in outcomes, such as mortality, with robust statistical power. Lastly, the observational nature of the study precludes definitive causal inferences, underscoring the need for prospective, controlled trials to substantiate best practices in diagnosing and treating fungal coinfections in COVID-19 patients.

## 5. Conclusions

Our findings underscore the considerable impact of fungal pulmonary coinfections on patients hospitalized with COVID-19. Despite sharing similar baseline comorbidities and demographic profiles with the COVID-19-only cohort, those with fungal involvement demonstrated amplified inflammatory responses, higher severity scores, and more frequent admissions to the ICU and use of mechanical ventilation. Although in-hospital mortality did not reach formal significance, the nearly doubled rate in coinfected individuals points to a clinically important trend. Microbiologically, a broad spectrum of fungal pathogens—ranging from *Aspergillus* and *Candida* species to *Pneumocystis*, *Cryptococcus*, and *Mucorales*—was observed, with notable rates of antifungal resistance that challenge conventional therapeutic regimens. Such findings emphasize the importance of systematic diagnostic evaluations, including bronchoalveolar lavage and prompt fungal culture or antigen-based assays, especially in patients with atypical disease progression or persistent elevations in inflammatory markers. Early intervention, informed by antifungal susceptibility results, may reduce morbidity and potentially curb mortality trends.

## Figures and Tables

**Table 1:** Demographics and comorbidities.

Parameter	COVID-19–Fungal (*n* = 64)	COVID-19-Only (*n* = 216)	*p*-Value
Age (years), mean ± SD	59.7 ± 9.8	58.5 ± 10.2	0.395
Male Sex, *n* (%)	37 (57.8)	127 (58.8)	0.869
BMI (kg/m^2^), mean ± SD	27.6 ± 4.7	27.3 ± 4.4	0.650
Hypertension, *n* (%)	36 (56.3)	121 (56.0)	0.974
Diabetes Mellitus, *n* (%)	19 (29.7)	64 (29.6)	0.996
Duration of Diabetes (years), mean ± SD	8.2 ± 3.3	8.0 ± 3.1	0.817
Chronic Kidney Disease, *n* (%)	10 (15.6)	29 (13.4)	0.657
COPD, *n* (%)	9 (14.1)	26 (12.0)	0.667
Malignancy, *n* (%)	6 (9.4)	14 (6.5)	0.428
Current or Ex-Smoker, *n* (%)	16 (25.0)	49 (22.7)	0.701
Days from Symptom Onset to Admission, mean ± SD	6.3 ± 1.9	5.8 ± 1.7	0.059
Corticosteroids, *n* (%)	45 (70.3)	110 (50.9)	0.004
Immunosuppressive Medications, *n* (%)	12 (18.8)	18 (8.3)	0.009
Specific Antifungal Regimens Used, *n* (%)	58 (90.6)	N/A	N/A
Azoles (*Aspergillus*, *Candida*)	20 (31.3)	N/A	N/A
Echinocandins (*Candida*, resistant strains)	18 (28.1)	N/A	N/A
Amphotericin B (*Mucorales*, severe cases)	12 (18.8)	N/A	N/A
Timing of Therapy Initiation (days post-admission), mean ± SD	2.3 ± 1.1	N/A	N/A

**Table 2:** Fungal species and major complications.

Fungal Pathogens and Complications	Frequency (*n* = 64)	Percentage (%)
**Fungal Species**		
*Aspergillus fumigatus*	20	31.3
Other *Aspergillus* spp.	4	6.3
*Candida albicans*	18	28.1
Other *Candida* spp.	8	12.5
*Cryptococcus neoformans*	5	7.8
*Pneumocystis jirovecii*	4	6.3
Mucorales (e.g., *Mucor*/*Rhizopus*)	4	6.3
Mixed Fungal Species	1	1.6
**Major Fungal Complications**		
Candidemia (Blood Culture Positive)	7	10.9
Aspergillus Invasive Pulmonary Infection	8	12.5
Mucormycosis with Tissue Invasion	3	4.7
Pneumocystis Pneumonia (clinical + PCR)	4	6.3
Cryptococcal Pneumonia (positive antigen/culture)	5	7.8
Candida Endophthalmitis	1	1.6

**Table 3:** Antifungal susceptibility and resistance patterns.

Organism	Total Isolates	Resistance to Azoles (%)	Resistance to Echinocandins (%)	Resistance to Amphotericin B (%)	Multi-Drug Resistance (%)
*Aspergillus fumigatus*	20	4/20 (20.0%)	-	-	2/20 (10.0%)
Other *Aspergillus* spp.	4	1/4 (25.0%)	-	-	1/4 (25.0%)
*Candida albicans*	18	2/18 (11.1%)	1/18 (5.6%)	0/18 (0.0%)	1/18 (5.6%)
Other *Candida* spp.	8	0/8 (0.0%)	2/8 (25.0%))	0/8 (0.0%)	2/8 (25.0%)
*Cryptococcus neoformans*	5	1/5 (20.0%)	N/A	0/5 (0.0%)	0/5 (0.0%)
*Pneumocystis jirovecii*	4	N/A	N/A	N/A	N/A
Mucorales	4	N/A	N/A	1/4 (25.0%)	1/4 (25.0%)
**Total/Overall Rate**	64	14.3%	9.5%	1.6%	9.5%

**Table 4:** Inflammatory markers and laboratory results.

Parameter	COVID-19–Fungal (*n* = 64), Mean ± SD	COVID-19-Only (*n* = 216), Mean ± SD	*p*-Value
C-reactive protein (mg/L)	85.7 ± 23.8	71.6 ± 20.4	<0.001
Procalcitonin (ng/mL)	2.4 ± 1.0	1.3 ± 0.6	<0.001
White blood cells (×10^9^/L)	8.7 ± 2.9	7.5 ± 2.4	0.003
Neutrophil-to-lymphocyte ratio	6.4 ± 2.3	4.9 ± 2.1	<0.001
Systemic immune inflammation index (×10^3^)	1185.3 ± 354.2	968.7 ± 316.9	<0.001
Platelets (×10^9^/L)	221.3 ± 67.2	236.6 ± 63.7	0.08
Albumin (g/L)	31.5 ± 4.9	34.0 ± 5.2	0.002

**Table 5:** Severity scores in COVID-19 patients with and without fungal infections.

Clinical Score	COVID-19–Fungal (*n* = 64), Mean ± SD	COVID-19-Only (*n* = 216), Mean ± SD	*p*-Value
APACHE II	18.6 ± 4.1	14.8 ± 4.0	<0.001
CURB-65	2.9 ± 1.1	2.2 ± 1.0	0.001
NEWS	8.4 ± 2.3	6.5 ± 2.3	<0.001

**Table 6:** Clinical outcomes in COVID-19 patients with and without fungal infections.

Outcome	COVID-19–Fungal (*n* = 64)	COVID-19-Only (*n* = 216)	*p*-Value
ICU Admission, *n* (%)	25 (39.1)	43 (19.9)	0.004
Mechanical Ventilation, *n* (%)	17 (26.6)	22 (10.2)	0.01
In-Hospital Mortality, *n* (%)	10 (15.6)	16 (7.4)	0.06
Length of Stay (days), mean ± SD	14.2 ± 5.4	11.5 ± 4.3	0.003

**Table 7:** Logistic regression for ICU admission.

Variable	Adjusted OR (95% CI)	*p*-Value
Fungal Coinfection	2.8 (1.5–5.2)	0.002
Age ≥ 65 years	1.9 (1.1–3.4)	0.02
APACHE II > 15	3.1 (1.7–5.7)	<0.001
Diabetes Mellitus	1.2 (0.6–2.1)	0.53
Male Sex	1.0 (0.6–1.8)	0.99
Antifungal Resistance	2.2 (1.2–4.0)	0.01

## Data Availability

The data presented in this study are available on request from the corresponding author.
